# Effects of Maternal and Cumulative Stress on Immune Phenotype and Stress Reactivity in Sows Later in Life

**DOI:** 10.3390/ani16101435

**Published:** 2026-05-08

**Authors:** Lily P. Hernandez, Alexis R. H. Main, Janeen L. Salak-Johnson

**Affiliations:** 1 Department of Animal and Food Sciences, Oklahoma State University, Stillwater, OK 74078, USA; lilphern@iu.edu (L.P.H.); alexismain@paradigmaag.com (A.R.H.M.); 2 Department of Large Animal Clinical Sciences, College of Veterinary Medicine, Michigan State University, East Lansing, MI 48824, USA

**Keywords:** stress response, immune function, life stress, cumulative experience

## Abstract

The timing and nature of gestational stress can alter offspring immune function and hypothalamic–pituitary–adrenal (HPA) axis regulation, yet the role of maternal stress history remains poorly understood. This study evaluated the effects of prenatal stress, maternal stress history, and repeated stress exposure on immune and stress reactivity in prenatally stressed (PS) gilts across two farrowings. Eleven PS second-parity sows (197.27 ± 19.22 kg) were sampled at multiple timepoints and compared with their maternally stressed (MS) dams. During the second pregnancy, PS sows received the same maternal treatment as their dams: placebo (CON) or hydrocortisone acetate (HCA) administered twice daily for 21 days during mid (M; d 51–72) or late (L; d 81–102) gestation. Data were analyzed using mixed models with repeated measures in SAS (Version 9.4). During the first pregnancy, PS gilts in the L-HCA group exhibited higher lymphocyte proliferation indices and plasma cortisol concentrations than MS dams (*p* < 0.05). In the second pregnancy, dehydroepiandrosterone sulfate concentrations were lower in PS sows in the M-HCA group than the CON sows (*p* < 0.05). Overall, cortisol concentrations were lower in PS sows than PS gilts (*p* < 0.05), suggesting that maternal stress history modifies physiological responses to gestational stress, though this interpretation is constrained by the small sample size.

## Simple Summary

This study sought to understand the effects of previous stress history and cumulative stress experiences on the ability of prenatally stressed sows to respond to maternal stress during two distinct periods of pregnancy. Results indicate that prior stress experiences influence immune and endocrine responses, with variations in cortisol levels, and lymphocyte proliferation indices contingent on gestational stage and treatment. Longitudinal tracking of prenatally stressed animals revealed that stress history and accumulated experiences can shape how an animal responds to subsequent stressors later in life. These findings highlight that an animal’s previous stress exposure can be as influential as the type and timing of a stressor in determining future stress responses. Therefore, it is important to consider both previous and cumulative stress experiences when assessing the long-term effects of prenatal stress in livestock.

## 1. Introduction

The exposure of the developing fetus to maternal stress during pregnancy, known as prenatal stress, has been studied extensively in humans and several animal models [1,2,3,4] with findings indicating that fetal exposure to stress can significantly impact physiological and behavioral development. Glucocorticoids play a crucial role in organ development and maturation; however, elevated maternal cortisol levels may disrupt these processes. Protective mechanisms, such as the placental enzyme 11β-hydroxysteroid dehydrogenase 2 (11βHSD2), are in place to prevent the fetus from being exposed to excessive maternal cortisol, while dehydroepiandrosterone (DHEA), an anti-glucocorticoid, counterbalances cortisol activity [5]. Under normal conditions, 11βHSD2 converts excess cortisol from its active form to its inactive form, cortisone [6,7,8]. However, during periods of chronic stress, the effectiveness of 11βHSD2 is diminished [9], thus exposing fetal tissues and organs to high levels of glucocorticoids. Under chronic stress conditions, DHEA and its inactive form dehydroepiandrosterone sulfate (DHEA-S) are shown to exhibit reduced anabolic and anti-glucocorticoid activity [10], allowing for further exposure to elevated levels of glucocorticoids. This exposure can result in progeny exhibiting unique phenotypes compared with those not subjected to prenatal stress. For example, sows that experience social stress during mid-to-late gestation gave birth to females that exhibited abnormal maternal behaviors (i.e., restless as piglets approached) [11].

Atypical phenotypes can develop due to multiple factors, including the timing of exposure and the type of stressor, but the outcomes can vary. Research indicates that even when stress occurs at the same stage of gestation, its consequences for progeny can vary greatly depending on the type of stressor. For instance, offspring born to dams that experienced heat stress during late gestation had higher cortisol levels and reduced weight gain than those dams not stressed [12], whereas repeated mixing of sows during late gestation did not lead to higher circulating cortisol concentrations [1]. These outcomes demonstrate that stressors exert distinct effects on the immune system and the hypothalamic–pituitary–adrenal (HPA) axis depending on exposure occur. Hence, the timing of exposure is critical to the phenotypic expression of prenatally stressed offspring, as the development and maturation of various systems span the entire period of gestation rather than a single period. The fetal immune system starts to develop in early gestation and continues through the last two-thirds of pregnancy [13]. Despite the expansive period of maturation, distinct periods of vulnerability may lead to different phenotypes. Kranendonk and colleagues [14] observed that giving oral hydrocortisone acetate (HCA) during early, mid, and late gestation resulted in increased play behavior in pigs born to sows stressed early, while those from the mid-stressed group exhibited longer fighting bouts. Administration of HCA resulted in a reduced humoral immune response in mid-born progeny and a delayed stress response in late-born ones when faced with an adrenocorticotropic hormone (ACTH) challenge [15]. Although research has advanced our understanding of how the timing and type of stressors affect prenatally stressed offspring, the effect of an animal’s lifetime stress experiences on shaping the responsiveness and phenotype of their progeny to future stressors, especially as breeding animals, remains largely unexplored.

Landrace–Yorkshire sows represent a common commercial maternal line who are selected for reproductive efficiency, including traits such as litter size and maternal performance, making them a representative of production-relevant trials. Studies have investigated the effects of prenatal stress on productivity and welfare in pigs, but most have focused on the immediate postnatal period [16,17,18,19]. Few studies have examined the long-term implications of prenatal stress for adulthood, and even fewer have examined its effects on the next generation [11]. Therefore, this study aimed to determine how cumulative life stress exposure influences immune and endocrine phenotypes in sows previously exposed to prenatal stress. Specifically, we sought to test whether observed outcomes were primarily driven by prenatal stress, subsequent maternal stress exposure during adulthood, or the combined effects of both across the lifespan. Outcomes were assessed using lymphocyte proliferation activity, adrenal steroid dynamics, and immune cell population profiling. Responses were evaluated longitudinally across developmental stages, including comparisons as gilts and later as sows, as well as relative to their dams.

A pharmacological model of prenatal stress was established using HCA administration during mid and late gestation to characterize immune and stress-responsive phenotypes of these animals. By integrating the effects of in utero stress at distinct gestational timepoints with comparable postnatal stress exposure, this approach provides a more comprehensive understanding of how prenatal stress shapes long-term physiological and immunological outcomes. Based on prior findings, mid-gestation exposure has been associated with a reduced humoral immune response, whereas late-gestation exposure has been linked to a delayed stress response. Based on these findings, we hypothesized that cumulative lifetime stress exposure, including re-exposure to a similar prenatal stressor, would exacerbate gestation-dependent differences in immune and endocrine phenotypes in prenatally stressed sows compared with non-prenatally stressed controls.

## 2. Materials and Methods

The experimental protocol was approved by the Oklahoma State University Animal Care and Use Committee (IACUC-22-62) and followed the Guide for the Care and Use of Agricultural Animals in Research and Teaching (Fourth Ed., 2020) [20]. All animals used in this study were bred, housed, and handled at the Oklahoma State University Swine Teaching and Research Facility (Stillwater, OK, USA).

### 2.1. Animals and Management

Eleven prenatally stressed (PS), second parity Landrace-Yorkshire sows inseminated with a single source Landrace semen source (DNA Genetics 241; 197.27 ± 19.22 kg) were used in a 2 × 2 factorial treatment design from day 30 ± 1 post-breeding through one week post-wean (30 ± 2 d post-farrow). Pregnancy was confirmed on day 30 ± 1 post-breeding using an ultrasound machine for trans-abdominal examinations (IBEX^®^ EVO^®^ II, E.I. Medical Imaging, Loveland, CO, USA). Sows were housed in individual gestation stalls (2.29 m × 0.61 m) and fed a standard gestation diet formulated to meet or exceed nutrient requirements according to the National Research Council (NRC; [21]). The amount of feed allotted was determined based on body condition score (BCS). A baseline amount of 2.3 kg was provided, and for every unit above or below the average BCS of 3, the amount fed was increased or decreased by 0.23 kg. Water was provided ad libitum via a water trough.

Five days before the expected farrowing date, sows were moved to the farrowing–lactation barn. They were housed in standard farrowing crates (1.5 m × 2.13 m) until weaning. At the time of birth, the entire litter was collected, dried, and weighed. All piglets in the litter were ear-tagged with unique numbers to identify birth order for ease of tracking. Trained technicians and graduate research assistants supervised all animals during farrowing, from the birth of the first piglet to the last in the entire group. After farrowing, sows and their piglets were checked twice daily until weaning. Sows were fed a standard lactation diet formulated to meet the NRC requirements [21]. Feed and water were offered ad libitum until weaning.

### 2.2. Experimental Design and Treatment

This study was conducted as a subset of a larger, funded study of the effects of prenatal stress across generations. The present objective focuses on immune and endocrine outcomes, and consequently, sample size and treatment distribution were limited, resulting in unequal sizes.

**Figure 1:**
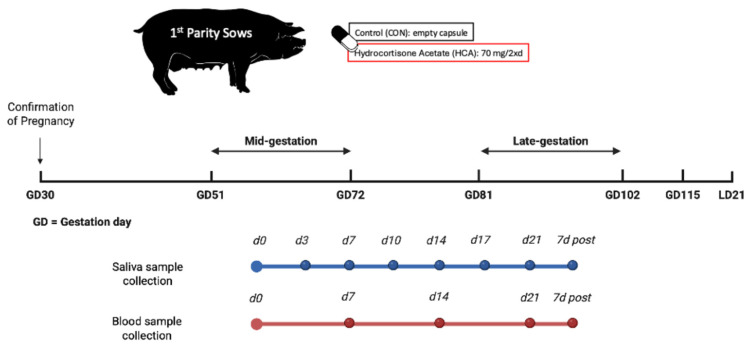
Schematic of the experimental timeline for maternal treatment and biospecimen collection.

After the PS-gilts farrowed, they were rebred and then received the same experimental treatment as their nulliparous dams—in other words, as 2nd-parity sows, they were assigned the same maternal treatment they experienced in utero. Treatments were either an empty gelatin capsule (CON) or a gelatin capsule containing 70 mg of powdered Hydrocortisone Acetate (HCA; Spectrum Chemical MFG Corp., Gardena, CA, USA) given during mid (M; Gestation Day 51 to 72) or late (L; Gestation Day 81 to 102) gestation, resulting in four treatment groups: M-CON (n = 2), L-CON (n = 2), M-HCA (n = 4) or L-HCA (n = 2). The dosage of HCA was determined based on the findings of Kranendonk et al. [22] and Mack et al. [23] where salivary and plasma concentrations increased in comparable measures to the concentrations detected in pregnant sows after psychologic stressors. Capsules were hand-fed at 0630 h (when feed dropped) and then again at 1830 h for 21 days. Acclimation to hand-feeding gelatin capsules began 3 days before the start of treatment, with sows fed empty gelatin capsules in a carrier substance that had no nutritional value. To minimize cross-contamination throughout the study, technicians wore gloves and changed between treatment groups, with the HCA treatment group being fed first, followed by the CON group (empty capsules). Figure 1 summarizes the study design.

### 2.3. Data Collections, Chemical Analysis, and Calculations

#### 2.3.1. Performance and Productivity Traits

Sow body weight was recorded at gestational day 30, before treatment, end of treatment, prior to farrowing, and at weaning. Litter traits, such as total born alive, litter weight, number of stillborn, and number of mummies, were recorded in real time. The total duration of parturition and the interval between each piglet born were also recorded during the farrowing process. Piglet body weight was recorded at birth and on lactational days 7, 14, 17, and 21.

#### 2.3.2. Sample Collection and Processing

Blood samples were collected prior to treatment, on days 7, 14, and 21 of treatment, and 7 days post treatment. Samples were collected via jugular venipuncture using 30 mL sterile syringes containing 4.5 mL of heparin solution and stored on ice until transferred to the lab. Blood was collected within ≤2 min of snaring to minimize increases in peripheral cortisol.

Saliva samples were collected on the same days as blood samples, with additional samples obtained on days 3, 10, and 17 of treatment, on gestational day 110, and at the onset of parturition. Samples were collected using a cotton swab (Salivette, Sarstedt Ag & Co., Nümbrecht, Germany), with one end zip-tied for ease of collection, as described by [24]. Animals were allowed to chew on the cotton swab for 1 min, the zip-tie was removed, the swab was placed in the tube, and the tube was placed on ice. Samples were then centrifuged at 2000× *g* for 30 min at 18 °C. Aliquots of the saliva sample were stored at −20 °C until analysis. It should be noted that samples collected during parturition were deemed “Quantity Not Sufficient” due to insufficient saturation of the swab, even when sows chewed for over 1 min.

A minimum of 200 μL of whole blood was used to measure total white blood cell (WBC) counts electronically with the Element HT5 Hematology Analyzer (Heska, Loveland, CO, USA). Ten mL of blood was transferred from the Vacutainer into a 50 mL conical tube (Corning, Tewksbury, MA, USA) and diluted 1:1 with Roswell Park Memorial Institute medium (RPMI; Gibco, Carlsbad, CA, USA). Diluted blood was layered over Histopaque-1077 (density = 1.077 g/mL; Sigma, St. Louis, MO, USA) and centrifuged at 700× *g* for 30 min at 25 °C. Lymphocytes were removed, washed twice in RPMI, counted with an automated cell counter (Countess III, Invitrogen Corp., Waltham, MA, USA) and adjusted to the appropriate cell concentration for each immune assay.

The remaining blood was then transferred to a 15 mL conical tube (Corning, Tewksbury, MA, USA) and centrifuged at 2100 RPM for 30 min at 4 °C. Plasma was collected and transferred to 0.5 mL microcentrifuge tubes (Thermo Fisher Scientific, Waltham, MA, USA) at −20 °C until analysis.

#### 2.3.3. Immune Assays

A non-radioactive cell proliferation assay kit (CellTiter96^®^, Promega, Madison, WI, USA) was used as described previously by [25], with minor modifications. Lymphocytes were used at a concentration of 5 × 10^6^ cells/mL and placed in triplicate in a sterile 96-well flat-bottom plate. Concanavalin A (Con-A) and lipopolysaccharide (LPS; Sigma, St. Louis, MO, USA) to stimulate T and B cells at a concentration of 0, 0.2, 2.0, and 20 μg/mL, 0, 0.5, 5, and 50 μg/mL, respectively. Plates were incubated for 68 h at 37 °C in a 5% CO_2_ humidified incubator, and then 20 μL of dye was added to each well. Plates were incubated for 4 h, and the reaction was stopped by adding 100 μL of stop solution to each well. Plates were read using a microplate reader (BioTek Epoch; Gen5 Version 3.04.17 Data Analysis Software; BioTek, Winooski, VT, USA) at 550 nm with a reference wavelength of 690 nm. Results are expressed as a proliferation index (P.I.):


$$P.I.=\frac{Optical\;Density\;(550/690\;nm)\;stimulated\;cells}{Optical\;Density\;(550/690\;nm)\;non-stimulated\;cells}\;$$

#### 2.3.4. Analysis of Endocrine Hormones

Cortisol, Cortisone, and DHEA-S were measured using a commercially available enzyme-linked immunosorbent assay (ELISA) following the manufacturer’s protocol (Arbor Assays, Ann Arbor, MI, USA). Plasma was diluted 1:100 for cortisol and cortisone, and 1:2 for DHEA-S, using the supplied dissociation reagent and assay buffer, and was run in duplicate. Saliva samples were diluted 1:10, and colostrum 1:200 in assay buffer. Plates were read using a microplate reader (BioTek Epoch; Gen5 Version 3.04.17 Data Analysis Software; BioTek, Winooski, VT, USA) at 405 nm. The intra- and inter-assay coefficient of variation for cortisol, cortisone, and DHEA-S were 4.18% and 4.61%, 3.22% and 4.10%, and 2.93% and 5.22%, respectively.

### 2.4. Statistical Analysis

All traits were tested for departures from normality using the UNIVARIATE procedure in SAS (Version 9.4, SAS Inst. Inc., Cary, NC, USA). A natural logarithmic transformation was applied to all traits that deviated from normality to facilitate the interpretation of results. Data were analyzed using the MIXED procedure in SAS, with sow as the experimental unit. Interactive effects of gestation stage, treatment, and gestation day were evaluated. The main effect of treatment was evaluated for the sow. Gestation day and treatment day were used as repeated-measures variables, with litter size as a covariate in the analysis of farrowing duration. During comparisons of maternally stressed (MS) gilts to PS gilts, the effect of generation (MS vs. PS gilts) and its interaction with treatment were evaluated where applicable. Given the study design, any comparisons between MS gilts and PS gilts inherently reflect differences in generation, age, and environmental timing (season), and therefore these factors are acknowledged as potential confounders in interpretation. For comparisons between PS gilts and MS sows, the interaction of treatment and cumulative stress experience was evaluated. Significant differences were reported at *p* < 0.05, and tendencies were reported when 0.05 ≤ *p* ≤ 0.10. Post hoc comparisons among treatment least-square means were adjusted for multiple testing using the Tukey–Kramer method.

## 3. Results

Since no samples were collected for parity comparisons, data are presented to assess how prior stress experiences shape sow responses to maternal stress (HCA treatment) during her second pregnancy. Thus, untreated PS-gilts were first compared with their MS-dams (including those receiving either HCA or control capsules) to evaluate the effects of prenatal stress and common, unavoidable stressors (i.e., mixing with unfamiliar animals) on immune phenotype and stress responsiveness during their 1st gestation (Section 3.1). Secondly, farrowing parameters were compared between PS-females during their first gestation with those of their second parity after they received the same treatment they were exposed to in utero to assess potential cumulative stress experiences on reproductive performance (Section 3.2). Thirdly, comparisons between maternal stress and gestational stage were conducted to assess how stress experience shaped sow immune and stress responses (Section 3.3).

### 3.1. Comparison of Progeny from HCA-Treated Gilt Dams

Given the multifactorial differences between groups, including generation, age, and season, results in this section should be interpreted as combined effects of prior stress exposure within a commercial production setting rather than as isolated effects of stress exposure alone. Presented in Table 1 are the mean immune and endocrine values during gestation for MS-gilts and their PS-gilt progeny. When comparing dams to their gilt progeny, L-HCA-treated PS-gilts had higher T-cell proliferation indices (*p* < 0.05) and a tendency toward higher B-cell indices (*p* < 0.10) than their MS-gilt dams. The L-HCA-treated PS-gilts also had higher plasma cortisol levels (*p* < 0.05) while maintaining a lower cortisol-to-cortisone ratio (*p* < 0.05) compared with their gilt dams. M-HCA-treated MS-gilts had significantly lower concentrations of plasma cortisol compared to PS-gilts (*p* < 0.05). Gilt dams and progeny of the CON-treated group were compared to determine whether the measured effects were due to prenatal stress exposure or to stressors experienced following birth (Table 1). Interestingly, PS-gilts exhibited a higher T-cell proliferation index (*p* < 0.05) than MS-gilts, while exhibiting a lower cortisol-to-cortisone ratio (*p* < 0.05). This was similar to the outcomes observed with the L-HCA-treated gilts.

Comparing treatments within relation groups (Table 1), PS-gilts in the M-HCA-treated group had a lower T-cell proliferation index than L-HCA- and CON-treated PS-gilts (*p* < 0.05). As for MS-gilt dams, those in the CON-treated group tended to have a higher B-cell proliferation index compared to those in the L-HCA group (*p* < 0.10). However, CON-treated MS-gilts had a lower cortisol-to-cortisone ratio (*p* < 0.05).

### 3.2. Comparison of Prenatally Stressed Gilts (1st Pregnancy) and HCA-Treated Sows (2nd Pregnancy)

Table 2 shows the effects of prenatal stress on lymphocyte proliferation indices and endocrine measures in prenatally stressed gilts during their initial gestation, compared with their experience of maternal HCA treatment during the subsequent gestation as sows. No significant differences in mean T- and B-cell proliferation indices and plasma cortisone concentration were observed based on cumulative stress experience and parity (*p* > 0.10). A difference in cumulative stress experience and parity was observed for plasma cortisol, with M-HCA-treated and L-HCA-treated sows having lower concentrations (*p* < 0.05) than when they were gilts. Interestingly, gilts who experienced prenatal stress at mid-gestation have higher concentrations of plasma cortisol (*p* < 0.05) than CON gilts. This difference, however, did not persist when they became sows as no difference across maternal stress effects were observed (*p* > 0.10).

The stress experience of gilts during their first pregnancy, coupled with maternal HCA treatment during their second pregnancy, had a significant impact on the body weight of their piglets (Table 3). MS-sows of the M-HCA group tended to birth heavier piglets than they did as PS-gilts (*p* < 0.10). All other measures, including the average number of piglets born alive, stillborn, mummified, and total farrowing duration, were similar between stress experience and parity.

### 3.3. Effects of Prenatal and Maternal Stress on Immune and Stress Status

Number and percentages of neutrophils and neutrophil-to-lymphocyte ratio were reduced (*p* < 0.05) in maternally stressed sows during late-gestation (L-HCA) compared to their counterpart controls (L-CON; Table 4). In contrast, the percentage of lymphocytes was higher in those maternally stressed in late gestation (L-HCA) than in the L-CON ones. Sows of the M-HCA-treated group had higher neutrophil numbers in the periphery compared to their control counterparts (M-CON). Total white blood cell and lymphocyte counts did not differ during treatment, regardless of maternal HCA treatment and gestational stage at the time of treatment.

Mean endocrine measures during maternal HCA treatment for sows during their second pregnancy are summarized in Table 5. Plasma cortisol was lower (*p* < 0.05) for sows in the L-HCA-treated group compared to those in the L-CON group, with M-HCA-treated sows having a higher plasma cortisol concentration (*p* < 0.05) compared to M-CON. However, sows in the L-HCA group had greater mean salivary cortisol concentration (*p* < 0.05) than L-CON sows. Sows of the M-HCA-treated group retained higher cortisol concentrations in saliva (*p* < 0.05) compared to their control counterparts. Notably, the cortisol-to-DHEA-S ratio was highest (*p* < 0.05) in M-HCA sows than in M-CON, despite DHEA-S concentration not being impacted by treatment. Notably, those in the L-HCA treatment group had a lower ratio (*p* < 0.05) than their control counterparts (L-CON) during their second pregnancy. No differences in plasma cortisone, DHEA-S, or the cortisol-to-cortisone ratio were observed during the treatment period among groups. A discrepancy between plasma and salivary cortisol in L-HCA sows is shown in Table 5, where salivary cortisol concentrations were higher than plasma. The reason for this difference cannot be determined from the present data.

**Figure 2:**
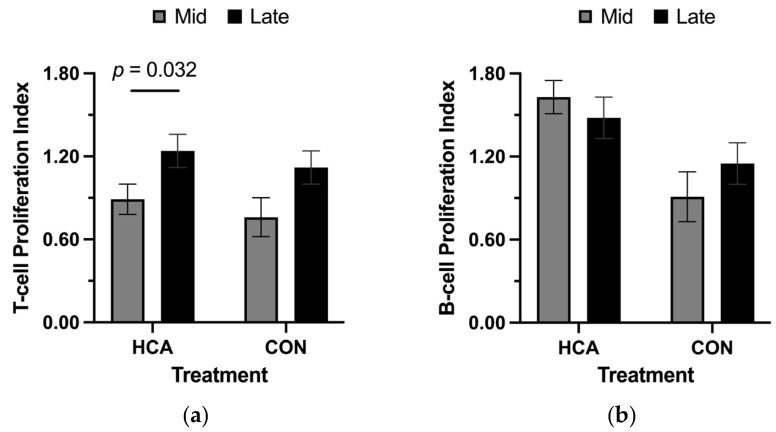
Effect of maternal treatment and gestational stage on mean lymphocyte proliferation indexes for (**a**) T-cells and (**b**) B-cells in prenatally stressed (PS) sows during treatment. Maternal treatments were hydrocortisone acetate (HCA) capsules or placebo (CON) fed for 21 days during mid gestation (M-HCA) or late gestation (L-HCA). Data are expressed as non-log-transformed means, with a *p*-value expressed as log-transformed.

Maternal HCA treatment influenced mitogen-induced lymphocyte proliferation, as shown in Figure 2. Sows in the M-HCA had lower mean T-cell proliferation indexes than those in the L-HCA treatment group (*p* < 0.05). Mean B-cell proliferation indexes were not impacted due to treatment and stage of gestation.

Table 6 summarizes the reproductive parameters and colostrum endocrine measures of sows across three treatment groups at farrowing and at the end of weaning. Maternal HCA treatment did not affect litter averages of total born, born alive, stillborns, mummies, and deads. Similarly, maternal HCA treatment did not influence the concentrations of colostrum cortisol, cortisone, or DHEA-S, or the cortisol-to-cortisone or cortisol-to-DHEA-S ratios. Total farrowing duration did not differ, nor did the time between piglets. Although piglet body weight did not differ at birth, sows in the M-HCA group weaned heavier piglets (*p* < 0.05) and tended (*p* < 0.10) to wean more kilograms of body weight per sow than CON sows.

## 4. Discussion

Prenatal stress has been shown to be associated with life-long alterations in immune and endocrine function that persist in adulthood. However, it is unclear how stress experienced during the lifetime of the progeny may further shape the outcomes related to prenatal stress exposure. In this study, we observed that the physiological phenotype of prenatally stressed (PS) animals may be further shaped by subsequent stress exposure, particularly when the stressor matches that experienced during in utero. This potential modulation of the prenatal stress phenotype is supported by the observations that M-HCA and L-HCA second-parity sows exhibit reduced cortisol concentrations compared with their gilt stage. Additionally, PS sows showed immune responsiveness when subjected to a similar stressor as their maternally stressed (MS) dams, suggesting that prior stress experiences may contribute to shaping subsequent physiological responses.

Comparisons between MS dams and their progeny confirm how stressful experiences, specifically prenatal stress, lead to altered phenotypes in immunity and stress responsiveness. It is proposed that the fetus alters its phenotype in anticipation of its new environment [26]. Given that PS gilts in both the M-HCA and L-HCA groups exhibited higher cortisol concentrations under “basal” conditions, it is theoretically possible that prenatal stress was associated with increased stress resilience in animals. One study found that men with higher basal levels of cortisol had reduced activation of the amygdala when subjected to stress [27]. It is interesting that the elevated plasma cortisol concentration did not persist once the progeny became sows and were exposed to maternal HCA treatment during their second pregnancy. This suggests that exposure to life stressors may continue to shape the stress phenotype of prenatally stressed offspring. The HPA axis regulates circulating cortisol levels during basal and stressful conditions, which is an attempt to minimize any harm to the organism [28]. However, focusing solely on cortisol captures only one aspect of the working state of the HPA axis. Cortisone, the inactive form of cortisol, can provide insight into the activity of the enzyme 11β-hydroxysteroid dehydrogenase 2 (11βHSD2), as it is known for converting cortisol to cortisone [6]. Although cortisone did not differ between MS gilts and PS gilts, and this also persisted when they became sows, the ratio of cortisol-to-cortisone tended to be lower in PS gilts. A lower ratio indicates less cortisol in circulation than cortisone, suggesting that animals are not in a state of stress. Once again, this raises the possibility that PS gilts may exhibit stress resilience related to prenatal exposure to maternal HCA treatment.

Beyond prenatal in utero priming, the altered response to maternal HCA treatment of the MS sows may indicate the role of later stress experience on shaping their phenotype. When these M-HCA females were prepubertal gilts and exposed to weaning and an ACTH challenge, they exhibited a normal stress response [15]. Although the exposure was acute, we did not observe a similar response in their salivary cortisol before maternal treatment, compared with that observed after 3 days of treatment. It is possible that the exposure to other stressors heightened their stress response. Results of this study agree with outcomes of PS rodent studies, which found that progeny exhibit a different response from their dam to the same stressor [29,30,31]. This suggests that exposure to maternal HCA treatment during development not only altered the phenotype of the progeny but also influenced how in utero exposure mediated responses to the same stimulus later in life.

Prior exposure to a stressor elicits an acute physiological response, but it also establishes a memory that shapes responses to future exposures [32]. As a result, subsequent responses to the same stressor are often less pronounced than the initial response. Novel stressors typically provoke a more robust response. Repeated exposure to one stressor can alter the responsiveness to a different stressor, a phenomenon termed cross-stress adaptation [33]. This proposed adaptation occurs where one stressor may enhance resilience or modify the responsiveness to other stressors. Plasma cortisol concentrations in PS females as gilts, compared with when they were sows, were significantly higher. This may show that the theory of cross-stress adaptation is present when exposed to maternal HCA treatment. Challenging PS females with a stressor while gilts, and again as sows, would allow for more concrete conclusions to be drawn. However, these findings suggest that stress alters cortisol secretion. Further studies, with the incorporation of stress challenges in both gilts and sows, are needed to confirm whether this concept of cross-stress adaptation occurs within PS MS sows.

A notable discrepancy was observed in the L-HCA sows, where salivary cortisol concentrations did not align with plasma cortisol. Under normal conditions, salivary cortisol is expected to parallel plasma cortisol [34]. However, in this study, cortisol was elevated in saliva relative to plasma. Given that plasma cortisol includes both bound and unbound fractions, while saliva primarily reflects the free fraction [34], this inconsistency is unlikely to be explained solely by differences in cortisol binding. One potential explanation may be the difference in sampling frequency. Saliva was collected every three days over the 21-day treatment period, whereas plasma samples were collected weekly. Although not presented, cortisol concentrations measured between blood sampling points appeared elevated in the L-HCA-treated sows. This suggests that weekly blood sampling may not have captured short-term increases in cortisol that occurred during the treatment period. Additionally, although HCA treatment was administered orally, saliva samples were consistently collected prior to capsule administration, minimizing the likelihood that sample contamination contributed to the observed differences.

The magnitude of immune and stress-related differences observed under maternal HCA treatment suggests that phenotypic alterations induced by prenatal stress may not become fully apparent until animals are re-exposed to conditions that recapitulate the prenatal stress environment. Regardless of the influence of life stress experience following prenatal stress, the phenotypic changes that occurred due to the timing of stress exposure in utero persisted. Sows exposed to maternal HCA treatment during mid-gestation exhibited immune-related phenotypes, including an upregulated humoral immune response. In contrast, those exposed during late gestation displayed phenotypes more closely associated with stress responsiveness. Specifically, during pregnancy, maternal cortisol concentrations and leukocyte populations typically change, including increases in neutrophils [35]. These changes are normal physiological adaptations that occur to support fetal growth. This is confirmed when evaluating the number of neutrophils in the periphery of L-HCA-treated sows compared to M-HCA-treated sows. It is well established that stress and HPA-axis hormones can modulate the immune response, either as enhancers or inhibitors [16,36,37,38,39]. L-HCA-treated sows exhibited an enhanced T-cell proliferation index compared to M-HCA-treated sows while possessing a significantly higher salivary cortisol concentration. This concept is highly plausible, as alterations in the stress response in L-HCA sows may drive a more enhanced rather than suppressed response during chronic stress. Overall, these outcomes highlight the importance of considering prior stress exposure in understanding its effects on immune function.

In addition to cortisol and cortisone, DHEA-S counteracts cortisol’s effects, acting as an immune stimulator to combat cortisol’s immune-dampening effects [40] in the M-HCA-treated sows during their second pregnancy. Although the concentration of DHEA-S was not different, the ratio of cortisol-to-DHEA-S was significantly higher in M-HCA-treated sows. This elevated ratio suggests that sows in the M-HCA group could be experiencing chronic stress [40], as cortisol levels are higher than DHEA-s. A larger sample size would be needed to confirm this assumption, however. In humans, it has been reported that individuals with high chronic stress burdens exhibit a similar pattern [10], suggesting that this may be a result of an elevated stress state sustained by chronic exposure. As the adrenal glands produce DHEA-S, chronic stress may prevent proper activation of the negative feedback loop, leading to a decline in DHEA-S concentration. Although not statistically different, L-HCA sows displayed a lower ratio than controls, suggesting that the stressor may not have induced a chronic stress state. This pattern may reflect a delayed stress response in the L-HCA group, as indicated by their plasma cortisol concentrations relative to those of their gilt dams. These findings imply that in utero stress exposure, when combined with subsequent life stressors, can differentially shape stress-response patterns in M-HCA versus L-HCA sows.

Cumulative life experience may have long-term implications on sow wellbeing, but not productivity, in response to maternal stress. Farrowing performance of PS sows following maternal stress was not altered compared to control, suggesting that future litters may be comparable to their non-stressed counterparts. Although wean weight was heavier in sows exposed to both prenatal and maternal stress compared to control sows, we cannot determine whether this effect is attributable to prior life stressors or other contributing factors. The long-term reproductive consequences of these phenotypes remain unknown, as sows were not followed beyond one week post wean. Outcomes such as return to estrus, non-productive days, and herd retention were not assessed and are considered important endpoints for future studies. In addition, while differences in stress responsiveness and immune phenotypes were observed, their functional relevance to animal health and welfare cannot be determined in the absence of a targeted immune challenge. Therefore, it remains unclear whether these phenotypes reflect adaptive resilience or increased vulnerability under commercial stress conditions.

A major limitation of this study is the small sample size and unbalanced distribution of animals across treatment groups. As this work is part of a larger longitudinal trial, the number of pregnant PS sows available for inclusion, as well as their allocation across treatment, were inherently limited. Animals were kept under standardized conditions, including housing, genetics, and feeding, which helped reduce environmental variation that can add noise to stress response data. Even with these limitations, the immune and endocrine responses observed in PS sows exposed to maternal HCA treatment were consistent with previously reported findings when they were prepubertal gilts [15]. This supports the idea that stress-related physiological changes may persist over time and may influence how animals respond later in life. Similar endocrine and immune outcomes have been reported in other swine stress models using different approaches to induce chronic stress. For example, repeated adrenocorticotropic hormone (ACTH) administration during mid gestation has been shown to increase plasma cortisol concentrations relative to controls [41], consistent with the elevated cortisol responses observed in the present study. Comparable increases in cortisol have also been reported in late gestation following the same model [42], aligning with the elevated salivary cortisol observed in L-HCA sows. In addition, social stress models have demonstrated increased cortisol secretion and altered immune responses, including higher T-cell proliferation [43], which is partially consistent with the immune responses observed here. Overall, while the small sample size limits statistical power, the consistency across outcomes and alignment with previous literature support these findings as biologically relevant and hypothesis-generating. These results contribute to understanding how prenatal stress history may shape later immune and endocrine function, and they highlight the need for larger, more balanced studies to further define these relationships in swine.

## 5. Conclusions

Despite the considerably small sample size and unequal distribution of animals, this study is among the first to explore how an animal’s stress experience shapes its subsequent physiological and phenotypic immune responses. By comparing MS gilts with their progeny as PS gilts, we observed that prenatal stress induces measurable phenotypic changes. As PS offspring matured into sows, subtle shifts in previously observed lactation traits emerged, indicating that early-life stress experiences, and potentially their cumulative effects, may influence responses to later challenges. Exposure to maternal HCA treatment may have induced gilts who were stress resilient, as reflected by elevated cortisol and cortisol-to-cortisone levels. However, when faced with a chronic stressor, this resilience was lost. Persistent differences in immune measures, including lymphocyte proliferation, further demonstrate the long-term impact of prenatal stress. As this experiment was part of a pilot study, running a replicate with a larger sample size with equal distribution of animals across treatments would further confirm our conclusions. Collectively, these findings emphasize that both stress history and cumulative effects are critical factors in evaluating the long-term consequences of prenatal stress in livestock.

## Figures and Tables

**Table 1:** Mean immune and endocrine measures of maternally stressed (MS) and prenatally stressed (PS) gilts during gestation ^1^.

	Timing of Stressor ^2^			
Item	M-HCA	L-HCA	CON	*p*-Value
T-cell Lymphocyte Proliferation Index				
MS Gilt	1.07 ± 0.07	0.82 ± 0.07 ^##^	0.81 ± 0.06 ^##^	0.001
PS Gilt ^3^	0.94 ± 0.07 ^b^	1.61± 0.07 ^#,a^	2.02 ± 0.11 ^#,a^	
B-cell Lymphocyte Proliferation Index				
MS Gilt	0.96 ± 0.17 ^xy^	0.55 ± 0.12 **^,y^	1.19 ± 0.10 ^x^	0.054
PS Gilt ^3^	1.13 ± 0.10	1.19 ± 0.12 *	1.08 ± 0.12	
Plasma Cortisol, ng/mL				
MS Gilt	15.95 ± 2.35 ^##^	10.93 ± 2.35 ^##^	14.16 ± 1.66	0.002
PS Gilt ^3^	23.21 ± 1.18 ^#^	22.47 ± 2.35 ^#^	17.62 ± 2.35	
Plasma Cortisone, ng/mL				
MS Gilt	-	20.56 ± 3.11	17.28 ± 2.20	0.317
PS Gilt ^3^	17.88 ± 1.56	17.66 ± 3.11	11.01 ± 3.11	
Cortisol-to-Cortisone Ratio, ng/mL				
MS Gilt	-	1.92 ± 0.20 ^#,a^	1.29 ± 0.14 ^#,b^	0.002
PS Gilt ^3^	0.79 ± 0.10	0.84 ± 0.20 ^##^	0.63 ± 0.20 ^##^	

^1^ Timepoints evaluated were gestational days 45 to 51 (mid) and 75 to 81 (late). ^2^ Maternal treatments were hydrocortisone acetate (HCA) capsules or placebo (CON) fed twice daily for 21 days during mid- (M-HCA) or late gestation (L-HCA). ^3^ PS gilts did not receive treatment during their first gestation. Cortisol-to-cortisone ratio not reported for M-HCA MS-gilts due to insufficient cortisone measurements (n = 1), preventing group comparisons. Within columns, means with different symbols ^#,##^ differ at *p* ≤ 0.05, and symbols *^,^** differ at 0.05 < *p* ≤ 0.10. Within rows, means with different letters ^a,b^ differs at *p* ≤ 0.05, and letters ^x,y^ differ at 0.05 < *p* ≤ 0.10.

**Table 2:** Mean lymphocyte proliferation indexes, cortisone, and cortisol measures for prenatal-stressed (PS) gilts and PS-gilts that were also maternally stressed (MS) as sows ^1,2^.

	Maternal Treatment ^3^			
Item	M-HCA	L-HCA	CON	*p*-Value
T-cell Lymphocyte Proliferation Index				
PS Gilt	0.92 ± 0.30	1.43 ± 0.30	1.00 ± 0.30	0.507
PS Sow	0.82 ± 0.25	1.39 ± 0.30	0.75 ± 0.30	
B-cell Lymphocyte Proliferation Index ^4^				
PS Gilt	1.38 ± 0.37	1.62 ± 0.37	0.94 ± 0.37	0.311
PS Sow	1.14 ± 0.30	3.36 ± 0.52	0.85 ± 0.32	
Plasma Cortisol, ng/mL				
PS Gilt	23.91 ± 1.56 ^#,a^	21.05 ± 2.21 ^#,ab^	12.27 ± 2.34 ^b^	0.001
PS Sow	11.08 ± 2.70 ^##^	3.76 ± 3.82 ^##^	13.04 ± 1.91	
Plasma Cortisone, ng/mL				
PS Gilt	17.79 ± 1.93	17.66 ± 3.35	11.01 ± 3.35	0.207
PS Sow	16.00 ± 2.73	8.89 ± 3.35	12.48 ± 2.37	

^1^ Comparing non-MS PS females in their first farrowing to the same animals as MS PS sows in their second farrowing. ^2^ Two timepoints were evaluated: gestational days 45–51 (mid gestation) and 75–81 (late gestation). Comparisons were conducted within each timepoint using measurements collected during the corresponding window. ^3^ Maternal treatments were hydrocortisone acetate (HCA) capsules or placebo (CON) fed twice daily for 21 days during mid-gestation (M-HCA) or late-gestation (L-HCA). ^4^ Data are expressed as non-log-transformed means but with log-transformed *p*-values. Within columns, means with different symbols ^#,##^ differ at *p* ≤ 0.05. Within rows, means with different letters ^a,b^ differ at *p* ≤ 0.05.

**Table 3:** Reproductive performance at farrowing of prenatally stress (PS) gilts and PS maternally stress (MS) sows ^1^.

	Maternal Treatment ^2^			
Item	M-HCA	L-HCA	CON	*p*-Value
No. of sows				
PS Gilt	4	3	4	
MS Sow	4	2	4	
Litter, average				
Born Alive				
PS Gilt	9.75 ± 2.13	11.00 ± 2.46	13.75 ± 2.13	0.350
MS Sow	15.50 ± 2.13	11.00 ± 3.01	15.25 ± 2.13	
Stillborn				
PS Gilt	0.00 ± 0.24	0.33 ± 0.28	0.25 ± 0.24	0.878
MS Sow	0.25 ± 0.24	0.50 ± 0.34	0.25 ± 0.24	
Mummies				
PS Gilt	0.75 ± 0.40	0.33 ± 0.46	1.25 ± 0.40	0.348
MS Sow	0.25 ± 0.40	0.50 ± 0.56	0.00 ± 0.40	
Piglet Body Weight, Kg				
PS Gilt	1.55 ± 0.10 *	1.48 ± 0.11	1.30 ± 0.10	0.077
MS Sow	1.23 ± 0.10 **	1.35 ± 0.14	1.13 ± 0.10	
Farrowing Performance				
Duration ^3^, Minutes				
PS Gilt	152.0 ± 84.3	317.0 ± 126.4	410.3 ± 103.2	0.628
MS Sow	160.5 ± 126.4	212.0 ± 126.4	233.0 ± 126.4	
Interval ^3^, Seconds				
PS Gilt	770.0 ± 350.1	1571.5 ± 428.8	989.5 ± 428.8	0.671
MS Sow	566.5 ± 428.8	1114.0 ± 428.8	790.0 ± 428.8	

^1^ Comparing non-MS PS females in their first farrowing to the same animals as MS PS sows in their second farrowing. ^2^ Maternal treatments were hydrocortisone acetate (HCA) capsules or placebo (CON) fed twice daily for 21 days during mid-gestation (M-HCA) or late-gestation (L-HCA). ^3^ Data are expressed as non-log-transformed means but with log-transformed *p*-values. Within columns, means with different symbols *^,^** differ at 0.05 < *p* ≤ 0.10.

**Table 4:** Effect of treatment and stage of gestation on mean leukocyte numbers and percentages in prenatally stressed (PS) sows during 21-day treatment period ^1^.

	Mid		Late		
Item	HCA	CON	HCA	CON	*p*-Value
White Blood Cells ^2^, 10^3^	10.29 ± 0.66	8.30 ± 0.90	10.37 ± 0.83	10.82 ± 0.83	0.171
Neutrophils, 10^3^	4.32 ± 0.33 ^abc^	3.12 ± 0.45 ^abd^	4.37 ± 0.41 ^b^	5.70 ± 0.41 ^a^	0.003
Lymphocyte, 10^3^	4.49 ± 0.34	3.95 ± 0.46	4.47 ± 0.42	3.91 ± 0.42	0.608
Neutrophil-to-Lymphocyte ^2^	1.00 ± 0.09 ^ab^	0.86 ± 0.13 ^ab^	1.00 ± 0.12 ^b^	1.60 ± 0.12 ^a^	0.002
Neutrophil, %	41.53 ± 2.00 ^ab^	38.20 ± 2.74 ^ab^	42.36 ± 2.50 ^b^	53.90 ± 2.50 ^a^	0.001
Lymphocyte, %	44.18 ± 1.63 ^ab^	47.14 ± 2.25 ^ab^	43.41 ± 2.05 ^a^	35.20 ± 2.05 ^b^	0.003

^1^ Maternal treatments were hydrocortisone acetate (HCA) capsules or placebo (CON) fed for 21 days during mid-gestation (M-HCA) or late-gestation (L-HCA). ^2^ Data are expressed as non-log-transformed means but with log-transformed *p*-values. Within rows, means with different letters ^a–d^ differ at *p* ≤ 0.05.

**Table 5:** Effect of maternal treatment and gestational stage on mean endocrine hormone levels in prenatally stressed (PS) sows during the 21-day treatment period ^1^.

	Mid		Late		
Item	HCA	CON	HCA	CON	*p*-Value
Plasma Cortisol, ng/mL	11.68 ± 1.05 ^abc^	7.33 ± 1.17 ^abd^	10.04 ± 1.40 ^b^	17.44 ± 1.35 ^a^	0.002
Salivary Cortisol ^2^, ng/mL	15.73 ± 3.17 ^abc^	5.78 ± 3.83 ^abd^	22.90 ± 4.23 ^a^	3.37 ± 3.83 ^b^	0.007
Plasma Cortisone ^2^, ng/mL	14.46 ± 0.98	13.97 ± 2.21	11.14 ± 1.44	12.36 ± 1.43	0.322
Plasma DHEA-S ^2^, ng/mL	7.39 ± 2.67	19.03 ± 3.69	5.31 ± 3.12	6.43 ± 3.12	0.212
Cortisol-to-Cortisone Ratio ^2^, ng/mL	0.74 ± 0.18	0.70 ± 0.33	1.34 ± 0.38	1.16 ± 0.25	0.481
Cortisol-to-DHEA-S Ratio ^2^, ng/mL	7.51 ± 4.42 ^a^	0.41 ± 5.57 ^b^	1.74 ± 4.87 ^ab^	13.93 ± 5.68 ^ab^	0.028

^1^ Maternal treatments were hydrocortisone acetate (HCA) capsules or placebo (CON) fed for 21 days during mid gestation (M-HCA) or late gestation (L-HCA). ^2^ Data are expressed as non-log-transformed means but with log-transformed *p*-values. Within rows, means with different letters ^a–d^ differ at *p* ≤ 0.05.

**Table 6:** Effect of maternal treatment and stage of gestation on the reproductive performance and colostrum endocrine measures of prenatally stressed (PS) sows.

	Maternally Stressed Treatment ^1^			
Item	M-HCA	L-HCA	CON ^2^	*p*-Value
No. of sows	4	2	4	
Litter, average				
Total Born	16.00 ± 2.10	11.50 ± 2.97	15.50 ± 2.10	0.476
Born Alive	15.50 ± 2.09	11.00 ± 2.95	15.25 ± 2.09	0.455
Stillborn	0.25 ± 0.27	0.50 ± 0.38	0.25 ± 0.27	0.843
Mummies	0.25 ± 0.21	0.50 ± 0.30	0.00 ± 0.21	0.422
Deads ^3^	0.50 ± 0.25	0.00 ± 0.35	0.75 ± 0.25	0.287
Farrowing				
Duration, Hours	2.68 ± 0.91	3.54 ± 0.91	3.88 ± 0.91	0.669
Interval, Minutes	9.45 ± 2.33	18.60 ± 2.33	13.15 ± 2.22	0.147
Colostrum Endocrine Hormones				
Cortisol, ng/mL	25.14 ± 5.70	29.43 ± 6.98	36.87 ± 5.70	0.408
Cortisone, ng/mL	41.72 ± 7.03	54.67 ± 8.61	52.13 ± 6.09	0.461
DHEA-S ^6^, ng/mL	504.58 ± 127.47	384.00 ± 180.27	549.13 ± 127.47	0.777
Cortisol-to-Cortisone Ratio	0.61 ± 0.17	0.54 ± 0.21	0.79 ± 0.17	0.635
Cortisol-to-DHEA-S Ratio	0.06 ± 0.03	0.08 ± 0.04	0.10 ± 0.03	0.697
Piglet Body Weight ^5^, Kg				
Kg birthed/sow	19.85 ± 2.20	15.35 ± 3.11	16.50 ± 2.20	0.445
Birth	1.23 ± 0.10	1.35 ± 0.14	1.13 ± 0.10	0.463
Kg weaned/sow ^6^	78.21 ± 9.71	72.40 ± 13.72	45.86 ± 9.71	0.315
Wean ^6^	8.25 ± 0.45 ^a^	6.58 ± 0.70 ^ab^	5.19 ± 0.50 ^b^	<.0001
Wean, Average				
No. Weaned ^6^	12.75 ± 0.87	11.00 ± 1.24	10.75 ± 0.88	0.292
No. Died ^4^	2.50 ± 1.10	0.00 ± 1.56	3.50 ± 1.10	0.252

^1^ Maternal treatments were hydrocortisone acetate (HCA) capsules or placebo (CON) fed for 21 days during mid gestation (M-HCA) or late gestation (L-HCA). ^2^ CON encompasses sows fed placebo capsules during mid or late-gestation. ^3^ Deads include piglets that were alive at birth but died shortly after due to aspiration or other causes. ^4^ Number of animals that died 1 day following birth up to weaning; this includes those found dead or euthanized. ^5^ Birth weight includes stillborn weights).^6^ Data are expressed as non-log-transformed means but with log-transformed *p*-values. ^a,b^ Means with different superscripts differ at *p*-value ≤ 0.05.

## Data Availability

Data presented in this study are available upon request from the corresponding author.
